# Novel Riboflavin-Inspired Conjugated Bio-Organic Semiconductors

**DOI:** 10.3390/molecules23092271

**Published:** 2018-09-05

**Authors:** Jan Richtar, Patricie Heinrichova, Dogukan Hazar Apaydin, Veronika Schmiedova, Cigdem Yumusak, Alexander Kovalenko, Martin Weiter, Niyazi Serdar Sariciftci, Jozef Krajcovic

**Affiliations:** 1 Faculty of Chemistry, Materials Research Centre, Brno University of Technology, Purkyňova 118, 612 00 Brno, Czech Republic; xcrichtar@fch.vut.cz (J.R.); heinrichova@fch.vut.cz (P.H.); schmiedova.veronika@seznam.cz (V.S.); kovalenko.alx@gmail.com (A.K.); weiter@fch.vut.cz (M.W.); 2 Linz Institute for Organic Solar Cells (LIOS), Physical Chemistry, Johannes Kepler University Linz, Altenbergerstraße 69, 4040 Linz, Austria; Dogukan.Apaydin@jku.at (D.H.A.); Cigdem.Yumusak@jku.at (C.Y.); serdar.sariciftci@jku.at (N.S.S.)

**Keywords:** bio-inspired material, conjugated materials, flavins, biomimetic energy storage, oxygen evolution

## Abstract

Flavins are known to be extremely versatile, thus enabling routes to innumerable modifications in order to obtain desired properties. Thus, in the present paper, the group of bio-inspired conjugated materials based on the alloxazine core is synthetized using two efficient novel synthetic approaches providing relatively high reaction yields. The comprehensive characterization of the materials, in order to evaluate the properties and application potential, has shown that the modification of the initial alloxazine core with aromatic substituents allows fine tuning of the optical bandgap, position of electronic orbitals, absorption and emission properties. Interestingly, the compounds possess multichromophoric behavior, which is assumed to be the results of an intramolecular proton transfer.

## 1. Introduction

Natural and bio-inspired organic conjugated materials [1,2,3,4,5,6,7] have become a new wave. Given the sustainability, biocompatibility, non-toxicity, versatility and potentially low-cost of bio-organic bio-inspired materials, it is evident that such materials will be widely considered as candidates for future bio-organic semiconductor science and technology, such as ultra-thin electronic platforms for surgical and point-of-care devices, diagnostic implants [8,9,10,11], soft robotics [12,13], biodegradable electronics [14,15] and food packaging [16].

Flavins, a group of naturally occurring materials, are especially remarkable due to their properties and high versatility from a chemical point of view. They belong to the redox coenzymes, being able to react as efficient one or two electron redox systems [17] accompanied by proton transfer at the nitrogen atoms of diazabutadiene of the isoalloxazine moiety, which makes them suitable as a prosthetic groups for a variety of enzymatic redox reactions [18,19] occurring over a wide potential range (>500 mV) [20,21]. An intriguing example is the role of flavin in the respiratory electron transport chain where the electrons from the reduced form of flavin adenine dinucleotide (FADH_2_) are transported along the group of proteins situated in the inner mitochondrial membrane carrying out proton flux across the membrane. An electrochemically induced proton gradient is generated resulting in the formation of high-energy adenosine triphosphate (ATP) [22]. Consecutive recycling of the flavin redox centers is furnished by FAD reduction in the Krebs cycle of mitochondria. On the basis of the analogy with this electrochemical cellular metabolism, several flavin cofactors have been proposed as lithium and sodium storing electrodes in rechargeable batteries [23,24,25]. Molecular tailoring of the structure of flavins showed that the capability to act as an effective redox center during charge transduction is preserved also by the structurally simpler lumichrome, alloxazine (tautomers of the isoalloxazine moiety of the flavin cofactors) and lumazine [24]. Furthermore, a recent work indicated that even slight branching of the alloxazine with electron-donating groups, inducing the lowering of the reduction potential, should allow for further increases in battery voltage made thereof [26]. In addition, flavin-containing donor-acceptor structures [27,28] have also found application in molecular logic gates or molecular switches [29,30] and in photovoltaics [30]. The photophysical properties and redox potentials of flavins are determined by hydrogen-bonding [31,32], metal coordination [33,34], π–π stacking [35] or the local environment [36]. On the other hand, the influence of enlarging the π-conjugated system on the optoelectronic properties of flavin derivatives remains less investigated, although shift of the absorption maxima to a longer wavelengths and change of the redox properties of the chromophore and of the extinction coefficient are expected [37,38]. Indeed, it was shown that the enlarging of the fused π-system from the basic *N*10-butyl substituted flavin core to the pyrene-attached derivative was accompanied by progressive bathochromic shift in both the absorption (up to 550 nm) and emission (up to 628 nm) spectra and all π-extended derivatives showed intensive emission with quantum yields of up to 80% while they maintained their electrochemical redox behavior analogous to parent flavin [39]. Despite the aforementioned viability of advanced alloxazine and lumazine derivatives of flavin as the charge-carrier materials for organic electronics only relatively few reports [40,41,42,43] about optoelectronic behavior are out there.

In the present paper, we report on the properties of novel alloxazine and lumazine derivatives investigated by means of UV/VIS spectroscopy, quantum-chemical calculations, thermogravimetric analysis with the intention of providing a comprehensive overview. We selected and synthesized a series of 11 fused and non-fused aromatic alloxazine and lumazine derivatives. The elongation of the π-conjugated system would provide bathochromic shift in absorption and emission characteristics [44], influence the redox potentials [45], while fusing of the aromatic rings further planarizes the molecule, thus enhancing the π-conjugated overlap [45].

## 2. Results and Discussion

### 2.1. Synthesis

**Scheme 1:**
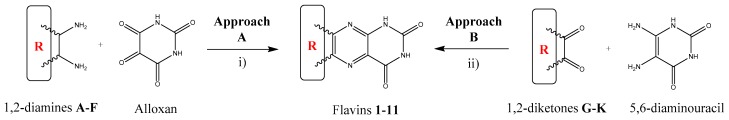
General scheme of the synthesis of flavins **1**–**11** by Approaches A and B. *Reaction conditions*: (i) AcOH, H_3_BO_3_; (ii) AcOH, MeOH/H_2_O/THF.

**Figure 1:**
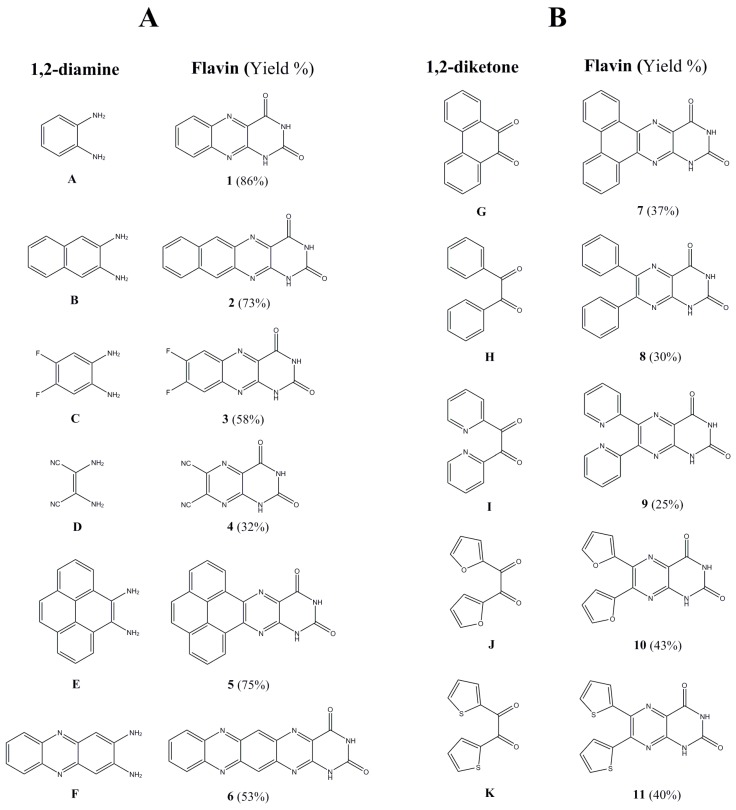
Compounds under investigation, synthetized by Approach A and Approach B.

The synthetic routes to the target compounds are shown in Scheme 1. As a result, using novel synthetic approaches, we synthetized a series of 11 alloxazine and lumazine derivatives. Even though the synthesis of compounds **1**–**4** and **6**–**11** was previously reported in the literature in varying detail [46,47,48,49,50,51], we envisaged two general and straightforward synthetic approaches (denoted as Approaches A and B), both of them differing in the source of the uracil moiety of the final flavin derivatives, improving the earlier procedures and providing the completely novel fused system **5**. The key step of the synthesis is represented by the formation of central pyrazine ring by the condensation of a suitable 1,2-diketone with a 1,2-diamine. Both approaches are complementary and enable the preparation of structurally advanced flavin derivatives (Figure 1) with relatively simple synthetic methodologies.

Approach A is the most favorable for the preparation of fused aromatic flavins from aromatic 1,2-diamines. It comprises the condensation of a 1,2-diamine with alloxan under neutral or acidic conditions (Scheme 1) [46]. The alloxan (monohydrate form) can be easily prepared by oxidation of barbituric acid with CrO_3_ in an acidic milieu of glacial acetic acid [52]. The flavins **1**–**4** were prepared from commercially available 1,2-diamines **A**–**D** (Figure 1) while the synthesis of the derivatives **5** and **6** required laboratory preparation of 1,2-diamines **E** and **F**. Pyrene-4,5-diamine **E** was prepared by three-step synthesis: oxidation [53] of pyrene by NaIO_4_ catalyzed by RuCl_3_ provided pyrene-4,5-dione (**L**) that was transformed to pyrene-4,5-dioxime (**M**) by reaction with NH_2_OH. **M** was subsequently reduced to **E** by catalytic hydrogenation using a H_2_/Pd/C system [54]. 2,3-Diaminophenazine **F** was obtained by the reaction of *o*-phenylenediamine **A** and FeCl_3_ in dilute hydrochloric acid [55]. We also probed the preparation of non-fused flavins by Approach A. It embodied finding a suitable means of synthesis of 1,2-diarylethane-1,2-diamines which could react with alloxan. Compared to aromatic 1,2-diamines, the reaction would provide 2,3-dihydropyrazines that would have to be oxidized to the final flavins [56]. We performed pilot experiments on the preparation of the flavin **8** with intermediate diamine (**P**) being prepared in a similar fashion to **E**. Benzil (**N**) was transformed to 1,2-dioxime (**O**) and submitted to the reduction. Contrary to the reduction of simple oximes to amines, reduction of 1,2-dioximes is probed in less detail. We tested several reduction methodologies used for the reduction of simple oximes, i.e., reduction by LiAlH_4_ [57], SnCl_2_/HCl [58], Red-Al [59], Pd/C/N_2_H_4_ [60], NaBH_4_/NiCl_2_ [61], NaBH_3_CN/TiCl_3_/NH_4_OAc [62] and NaBH_4_/TiCl_4_ [63] under various reaction conditions. Among them, only reduction with NaBH_4_/TiCl_4_ provided us with desired 1,2-diphenyl-ethane-1,2-diamine **P** with good yields. On the other hand, we could not obtain the desired 2,3-dihydropyrazine **Q** or directly flavin **8** by condensation with alloxan under either neutral or acidic glacial AcOH conditions. We have thus envisaged another approach.

Approach B is a suitable way to prepare non-fused aromatic flavins from 1,2-diketones and 5,6-diaminouracil, taking into consideration that from the synthetic point of view, 1,2-diketones are more readily available than the corresponding 1,2-diamines. The synthesis embodies a condensation of a 1,2-diketone with 5,6-diaminouracil under acidic conditions [64]. The procedure was improved by altering the solvent system by using of THF which dissolves the corresponding 1,2-diketones better than lower alcohols resulting in a broader spectrum of employable substrates. The intermediate 5,6-diaminouracil was obtained by a 3-step synthesis [65]. Condensation of ethyl cyanoacetate with urea under basic (NaOEt) conditions yielded 6-aminouracil. The nitrosation of 6-aminouracil provided 5-nitroso-6-aminouracil that was subsequently reduced to 5,6-diaminouracil by sodium hydrosulfite. Subsequently, the flavins **7**–**10** were prepared from commercially available 1,2-diketones **G**–**J** whereas the flavin **11** from the prepared diketone **K**. The 2,2′-thenil **K** was obtained by benzoin condensation of thiophene-2-carbaldehyde to 2,2′-thenoin and its in situ oxidation by ambient oxygen [66].

The synthesis of flavin derivatives by both approaches required appropriate purification methods in order to obtain high purity materials, suitable for further optical and electrochemical measurements. Due to very limited solubility of the all prepared compounds digestion in boiling methanol was used as the first stage of the purification. Vacuum sublimation was used as the final purification step. Target compounds with the final purity were used for further experiments.

The structures of the prepared flavins (except for compound **6** which is practically insoluble in common solvents) was confirmed by ^1^H-NMR and also by ^13^C-NMR in the case of compound **4** where only ^1^H-NMR could be considered insufficient (see Supporting Information). A typical phenomenon of the proton spectra for the target compounds is the appearance of two singlets in the 11–13 ppm region belonging to strongly deshielded N–H protons along with a downfield shift of the aromatic protons caused by their conjugation to the strongly electron-withdrawing diimide moiety. The purity of the final compounds was further checked by elementary analysis. Nevertheless, it has to be stated that additional quantitative and qualitative analysis of the purity of the final compounds by various either normal or reverse-phase HPLC methods proved to be rather inconclusive as the compounds are poorly soluble in all commonly employed solvents, including water. On the other hand, with the stepwise purification process involving digestion and vacuum sublimation we obtained final compounds with sufficiently high purity for the purposes of optical and electrochemical studies.

TGA measurements proved the high thermal stability of the flavin derivatives reaching 450 °C for compound **6**. The series of fused flavin derivatives (compounds **1**–**7**) showed a progressive increase of the decomposition temperature with the increasing number of fused aromatic rings (from 276.78 °C for compound **3** to 443.11 °C for the compound **6**, see Supporting Information). On the other hand, the series of non-fused flavin derivatives (compounds **8**–**11**) provided decomposition temperatures in the range of 305.62 °C (compound **10**) to 354.20 °C (compound **11**). It should be stated that the most of derivatives are stable to temperatures higher than 300 °C and compounds **5** and **6** even more than 400 °C. Such thermal stability, not often observed at small molecules, may strongly contribute to the suitability of the materials for the applications in organic electronics.

### 2.2. Optical Measurements

Flavin-like compounds are well known to possess the formation of tautomers in the solution by means of the proton transfer [67] resulting in the formation of the *iso*-form. Thus, in our case, the influence of the conjugated functional “tail” on the proton transfer would be noticeable in the absorption-emission optical spectra in DMSO. Depending on the electronegativity, conjugated length, planarity and the absence/presence of the free rotation we expect to observe clear differences in the optical behavior of the materials under study. Indeed, how it is shown below, the optical measurements of the considered compounds, possessed interesting, but complex behavior.

The optical spectra of all compounds under study consisted of more than one fluorescence band with different intensity, which on one hand can be an indication of impurities. Indeed a small amount of impurities can drastically affect the emission spectra, however on the other hand, the assumption that the multi-chromophoric behavior is based on the correspondence of the absorption spectra with the excitation ones. In this case the possible impurities, which could remain after the recrystallization should not affect the absorption spectra. Moreover, the formation of the tautomers in other alloxazine derivatives, i.e., lumichrome [67] or 6,7-dicyanolumazine [40] (compound **4**) are well known. As an example, it has been reported, that in aqueous solution of the 6,7-dicyanolumazine at neutral pH, it possesses two-chromophoric behavior with two different absorption bands at 350 and 400 nm. However in the case of the whole family of materials under study, it is practically impossible to perform the measurements in aqueous solution for all the derivatives, as most of the derivatives cannot be dissolved in water. However, it has to be noted, that due to the high purity of our compounds the second chromophore effects are unlikely to be caused by impurities and/or degradation products (see TGA and NMR spectra in the Supporting Information). Thus, considering the formation of the *iso*-form from the alloxazine derivatives, we presume the presence of two chromophores marked Ch. A and Ch. B. Moreover, the equilibrium concentration of tautomers can be changed by conditions like temperature or acidity of solvent. The effect of the environment of pH is common for the flavins, thus additionally we demonstrate the effect of acidity of the environment on the fluorescence emission spectra of the compound **1** (see Supporting Information). There is clear evidence that long wavelength emission of the chromophore (Ch. B) is missing in acidic environments in contrast to the basic ones, which indicates that the proton transfer is hindered and one of the tautomers is eliminated in the acidic solvent.

**Figure 2:**
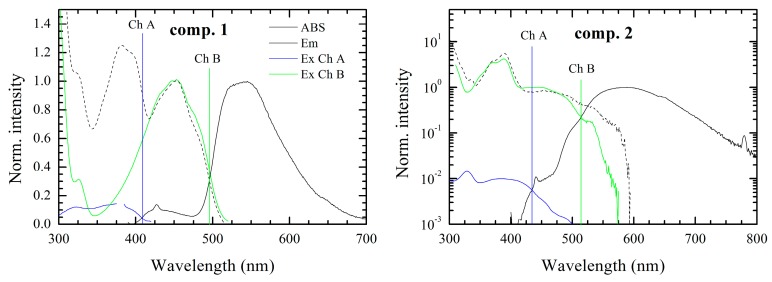

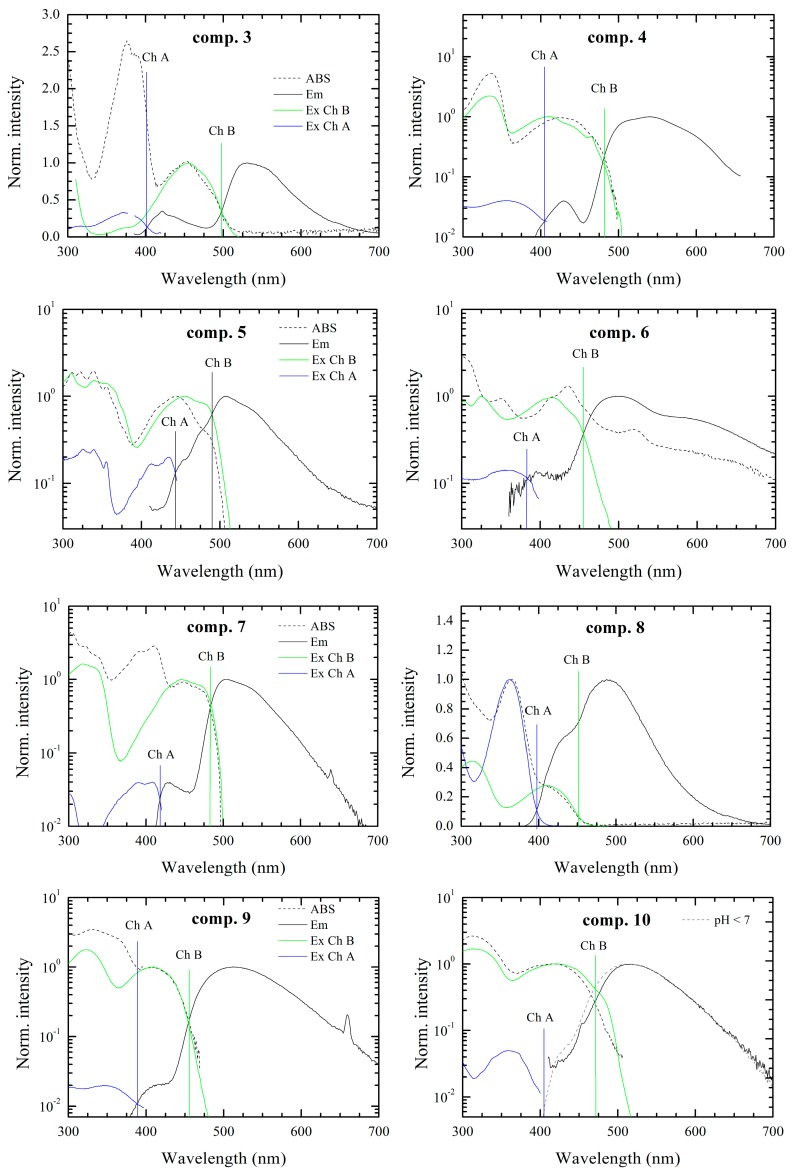

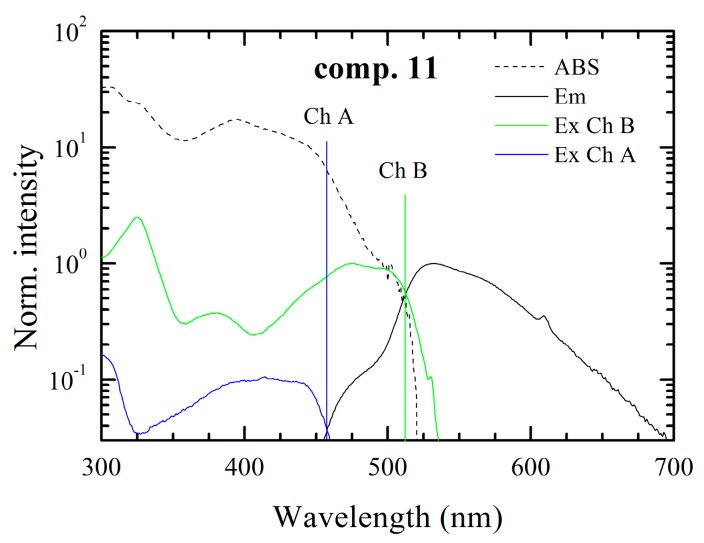
UV-Vis absorption, excitation and emission spectra of the compounds considering two-chromophore model with the marked optical band gaps of observed chromophores.

It has to be noted that the intensity ratio, in some cases differs by up to a few orders of magnitude (especially for compounds **2**, **4**, **7**, **9**). When, the emission band of the chromophore with higher energy gap (Ch. A) has smaller intensity due to reabsorption in all the cases, which results from spectral overlap between emission and absorption spectra as depicted in Figure 2. Simultaneously these spectral overlaps indicate a resonance energy transfer from the higher energy chromophore (Ch. A) to the lower energy chromophore (Ch. B), which amplifies the fluorescence intensity of chromophore Ch. B.

In the case of compounds **2**, **5**, **8**, **10**, **11**, the overlap between emission spectra of the observed two chromophores was seen. This situation makes the resolution of chromophores in spectra more speculative, but the presence of two chromophores was confirmed by measurement of the excitation spectra. If the emission spectrum were originated from only one chromophore the excitation spectrum would follow the absorption spectrum. However this condition is not fulfilled for the compounds under investigation. On the other hand, superposition of excitation spectra of Ch. A and Ch. B chromophores well fitted to the absorption spectrum in the solution.

Exception occurs for solution with compounds **6** and **10**. Seemingly, in case of compound **6** the appearance of a third chromophore can be observed. Moreover excitation and emission spectra attributed to chromophore Ch. B have resolved vibronic structures with overlap of zero-phonon peaks. This small Stokes shift is characteristic of very rigid structures with very small reorganization energy. The compound **10** in DMSO solution provides only one fluorescence band attributed to Ch. B according to its position. Nevertheless, in the acidic environment the emission band of Ch. A occurs. Thus, in neutral environment we presume that the fluorescence of Ch. A is effectively reabsorbed by and/or quenched by energy transfer to Ch. B.

**Figure 3:**
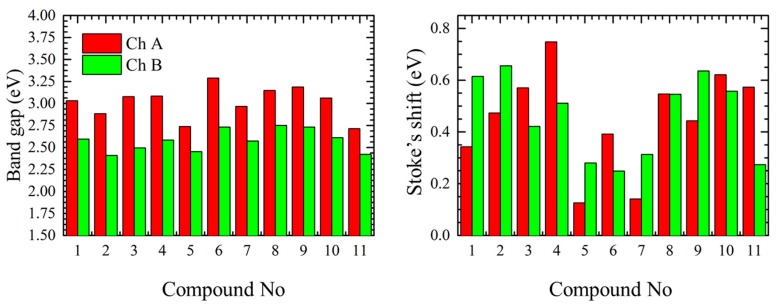
Optically measured bandgap and Stoke’s shift values for the compounds **1**–**11**.

The optical band gap values (Figure 3) were determined as the intersection point between normalized excitation (absorption) and emission spectra of each chromophore. Generally, the optical measurements have shown that the short-wavelength chromophores (Ch. A) have an absorption band in the range of 2.7–3.3 eV and the long-wavelengths chromophores (Ch. B) in the range from 2.4 to 2.8 eV. Therefore, the band gap difference between long- and short-wavelength chromophores is about 0.5 ± 0.1 eV for the most of the cases, however, in case of compounds **5** and **11** the difference of 0.3 eV was observed.

Values of Stoke’s shift (Figure 3 and Table 1) for the shorter linear compounds **1**–**4** were in the range of 0.3 to 0.8 eV for the alloxazine form and in the range from 0.4 to 0.7 eV for the *iso*-form. In this case the deviation of the Stoke’s shift is caused by the reorganization of the electron density along the linear molecule during the excitation depending on its length (e.g., compounds **1** and **2**) and/or the presence/strength of the electron withdrawing substitutions competing with the alloxan ending functional group (e.g., compounds **3** and **4**). In case of more complex structure, as for compounds **5**–**7**, we observed smaller Stoke’s shift values for both alloxazine and isoalloxazine forms, this can be explained that in case of planar non-linear rigid structures the reorganization energy is lower (compounds **5** and **7**) or the electron density is equally distributed due to the acceptor groups equilibrium (compound **6**) as it will be shown later. On the other hand the presence of the free rotation in the non-fused compounds **8**–**11** is resulting in relatively large Stoke’s shift.

Nevertheless, to understand the complex optical behavior of the materials we should take a deep insight into the molecular structures if the compounds. Compounds **1**–**7** are fused, having donor (**1**, **2**, **5**, **7**—benzene, naphthalene, pyrene and phenanthrene moiety, respectively) or acceptor (**3**, **4**, **6**—difluorobenzene, dicyano and phenazine moiety, respectively) “tails” anchored to the central pyrazine ring, similarly to non-fused compounds, having electron poor (**8** and **11**—phenyl and thiophene moieties, respectively) and electron rich (**9** and **10**—pyridine and furan moieties, respectively) conjugated groups. Nevertheless, it would be feasible, that the electronegativity of the conjugated part will affect the proton transfer. Thus, for materials, having both electron donating (fused aromatic rings for the materials **5** and **7**) and electron accepting (pyridine and furan for the materials **9** and **10**) we assume a multi-chromophore model and performed DFT modeling.

### 2.3. DFT Modeling

Geometry optimization was performed using the standard B3LYP functional expanded with the 6-311G** basis set, which has proved its accuracy for organic conjugated systems. The solvent effect was simulated using the SCRF procedure. The computational protocol was firstly to find the energy minima of the alloxazine form and then to optimize isoalloxazine derivative: direct comparison of the energy difference between the initial and *iso*-form is intended to give a general overview about the stability of the given tautomer. To get an overview about the electronic transitions the absorption spectra of the compounds in both alloxazine and isoalloxazine forms were calculated for the 10 lowest states. The comparison between the simulated absorption spectra of alloxazine and isoalloxazine tautomers has shown that the spectra of the *iso*-form are bathochromically shifted (see Supporting Information).

Regarding the energy difference between the initial and iso- form, the optimized structures were compared to each other, considering the compounds are dissolved in the DMSO. Theoretical approach has shown that in all the cases alloxazine form was found to be favorable. The lowest isomerization energies were found for the compounds **7**, **8**, **10** and **9** (0.02, 0.09, 0.10 and 0.16 eV respectively), for the compounds **1**, **2**, **3**, **5** and **6** the energy difference between the alloxazine and isoalloxazine form was in the range of 0.18 to 0.22 eV and the highest isomerization energy was computed for the compounds **4** and **11** (0.28 and 0.27 eV, respectively). Thus we assume that the alloxazine form is dominant, and as in the absorption spectra, the short-wavelength form is prevalent, however higher absorption of the chromophore does not indicate a higher ratio of the chromophore A. In the present case the ratio of the various chromophores will be highly affected by the solvent and needs further detailed study. Moreover we do not exclude that depending on the environment, additional chromophores can appear due to protonation/deprotonation, carbonyl group reduction, etc.

**Figure 4:**
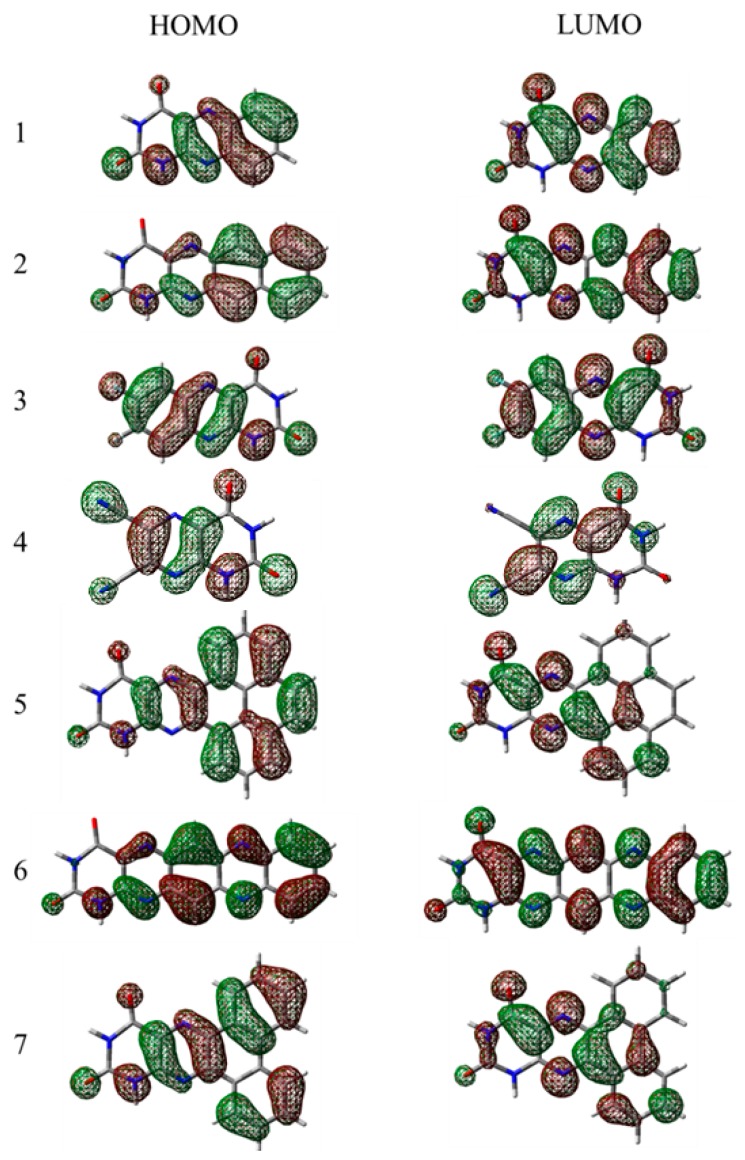
DFT calculated HOMO and LUMO orbitals of the fused alloxazine form compounds.

**Figure 5:**
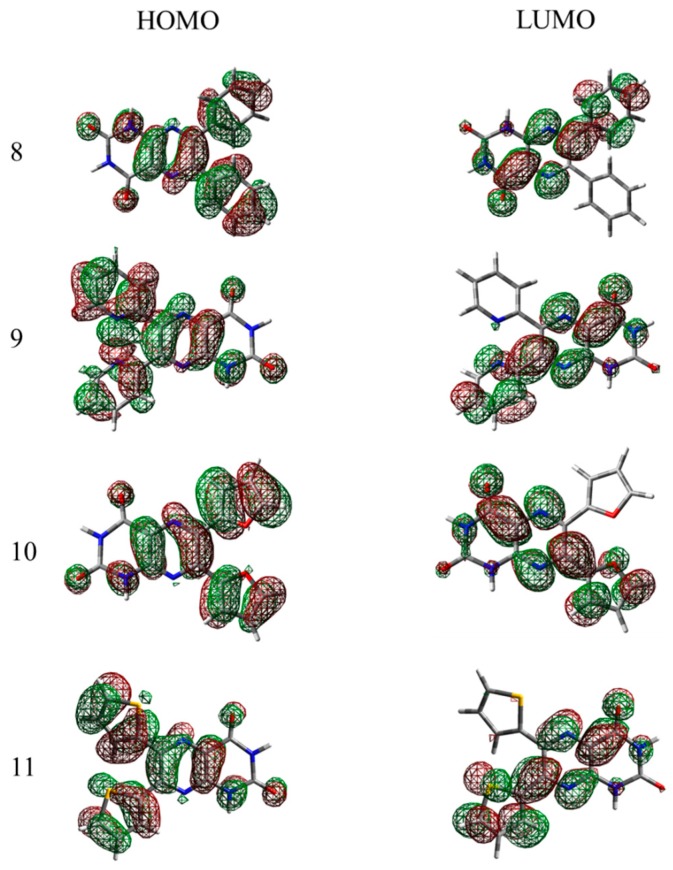
DFT calculated HOMO and LUMO orbitals of the non-fused alloxazine form compounds.

In Figure 4 and Figure 5, the HOMO and LUMO orbitals of the compounds are depicted (for the *iso*- form see the SI). All of the linear planar fused molecules were found to have a homogenous electron density distribution, which results in a relatively low band gap, unusual for molecules of such size, especially for compound **6**, where the presence of the electron-rich pyrazine building block results in a nearly homogeneous electron density distribution equally “stretched” along the molecule. In the case of non-linear fused compounds (molecule **5** and **7**) a slight asymmetry in the LUMO was observed—the electron density is delocalized on one of the aromatic rings, thus the optical gap for the bigger molecules e.g., **5** and **7** is similar to that of the smaller ones (e.g., **1**, **3** and **4**).

It has to be noted that like the non-linear fused compounds **5** and **7**, non-fused systems in the excited state (LUMO orbitals) possess an electron density selectively distributed near only one of the aromatic rings in the 7th position which, however, can be explained by the asymmetrical alloxazine group, where strongly electronegative carboxyl groups cause a redistribution of the electron density, resulting in unequal rotation angles between the alloxazine core and the non-fused aromatic rings. Generally, in the optimized ground state the dihedral rotation angles between the core and the aromatic substituents for the compounds **8**–**11** are stated in Table 2. Interestingly, the isomerization of the compound results in the redistribution of the electron clouds due to the interaction of the transferred proton in the position 8th and the aromatic rings, especially in the case of the non-fused compounds. This is the result of the free rotation between the isoalloxazine core and the abovementioned ending groups. Electropositive hydrogen in the *iso*-tautomers repulses the aromatic rings, especially with the electron-poor thiophene, resulting in the large difference in the dihedral angles of the two tautomers.

### 2.4. Electrochemical Measurements

Lumichrome derivatives usually exhibit a redox reaction associated with the transfer of two electrons and two protons, which is highly important when the application of such materials in high-energy-density redox-flow batteries is considered [68]. Almost all of the derivatives, except **3**, **8**, and **9**, showed promising electrochemical behavior in the positive part of the voltammogram which makes them possible candidates for catalytic reactions like oxygen evolution reaction conditions. Upon reduction, the compounds **1** and **8** showed reversible 2-electron reduction while **3** exhibited 1-electron reversible reduction. Compound **5** showed on the other hand, revealed 2-electron irreversible reduction behavior. These compounds might be suitable candidates for hydrogen evolution reaction in water. Table 3 shows the energy levels (HOMO and LUMO) of flavin derivatives calculated using electrochemical methods.

Comparing the electrochemical measurements with the values obtained from the optical measurements we can assume that the measured values are related to the low band-gap isoalloxazine form for the derivatives **1**, **3**, **4**, **5** and **11**. For compound **6** the band gap obtained by the electrochemical measurement was found to be significantly smaller than the one measured optically, and further measurements are needed to explain this phenomenon.

All of the compounds suffer from high solubility in organic solvents upon reduction and oxidation. Their electrochemical behavior in aqueous media, as well as their catalytic performance, is currently under investigation.

## 3. Conclusions

The novel synthesis approaches, and comprehensive characterization, including optics, DFT modeling and electrochemistry of various novel Flavin-inspired derivatives are described in the present paper. The compounds possess high thermal stability and chemical versatility, which indicates their potential as building blocks in the future (e.g., as copolymers or metal-organic complexes). Extension of the conjugated tail of the alloxazine group bathochromically shifts the absorption and emission spectrum. However, as was shown, in solution the compounds possess multi-chromophoric behavior due to proton transfer and the formation of the isoalloxazine form. The appearance of the tautomeric forms can be affected by many factors, including the molecular structure, solvent, pH, etc., which requires the comprehensive study of each individual compound. According to the electrochemical measurements the materials under investigation show promising electrochemical behavior for catalytic reactions like oxygen evolution. Moreover these compounds can be successfully applied as a redox centers in biomimetic energy storage [25].

## 4. Experimental Section

### 4.1. Materials and Synthesis

All solvents and reagents were obtained commercially and used as received unless stated otherwise. All moisture-sensitive reactions were performed in dry flasks fitted with glass stoppers or rubber septa under a positive pressure of argon. Air- and moisture-sensitive liquids and solutions were transferred by syringe or stainless steel cannula. Anhydrous Na_2_SO_4_ was used to dry organic solutions during workup, and evaporation of the solvents was performed under reduced pressure using a rotary evaporator. Flash column chromatography was performed using 220–440 mesh silica gel. Thin-layer chromatography was conducted on Supelco 60 TLC plates (Sigma Aldrich, St. Louis, MO, USA) with 254 nm fluorescent indicator. Spots were observed under UV irradiation (254 nm or 354 nm). ^1^H- and ^13^C-NMR spectra were recorded in CDCl_3_ or DMSO-*d*_6_ using an Avance III 300 MHz spectrometer (Bruker, Billerica, MA, USA) with working frequencies of 300 MHz and 75 MHz, respectively, at 30 °C. Chemical shifts are expressed in parts per million (δ scale) downfield from tetramethylsilane and are referenced to residual protons in the NMR solvent (CHCl_3_: δ 7.25 ppm, DMSO: δ 2.50 ppm). Coupling constants (*J*) are given in Hz with coupling expressed as s—singlet, bs—broad singlet, d—dublet, dd—doublet of doublet, t—triplet, tdd—doublet of triplet of doublets, ddd—doublet of doublet of doublets, m—multiplet. Elemental analysis was performed using an EuroEA3000 Elemental Analyser (Eurovector, Pavia, Italy). Melting points were determined using a Kofler apparatus equipped with a Nagema PHMK 05 microscope (Nagema, Dresden, Germany) and the temperatures were not corrected. Thermogravimetric analysis was performed using TGA Q50 instrument (TA Instruments, New Castle, DE, USA) with nitrogen as the carrier gas. Mass spectra were recorded on a GC–MS spectrometer ITQ 700 (DEP) (Thermo Fisher Scientific, Waltham, MA, USA) IR spectra were recorded on an ALPHA FT-IR Spectrometer (Bruker).

### 4.2. General Procedure for the Preparation of the Fused Flavins ***1**–**6*** by Approach A

The corresponding diamine (1.5 mmol, 1 eq.) was mixed with alloxan monohydrate (0.21 g, 1.5 mmol, 1 eq.) and boric acid (0.092 g, 1.5 mmol, 1 eq.) in glacial acetic acid (10 mL). The mixture was stirred under an Ar atmosphere at 60 °C for 15 h. The reaction mixture gradually turned into a suspension. The solid fraction was collected by filtration, washed with acetic acid (20 mL) and diethyl ether (20 mL). The crude product was purified by digestion in boiling methanol. Digested flavin derivative was further subjected to vacuum sublimation to yield the final target compound.

### 4.3. Benzo[g]pteridine-2,4(1H,3H)-dione *(**1**)*

Compound **1** was synthesized according to the General Procedure, Approach A. Reaction of diamine **A** (0.500 g, 4.62 mmol) with alloxan monohydrate (0.741 g, 4.62 mmol) and boric acid (0.291 g, 4.62 mmol) provided compound **1** as a light green solid (yield 0.56 g, 86%). M.p. > 330 °C (lit. >400 °C) [69]. ^1^H-NMR (DMSO-*d*_6_, TMS): δ 11.92 (s, 1H), 11.73 (s, 1H), 8.17 (d, *J* = 4.8 Hz, 1H), 7.96–7.91 (m, 2H), 7.81–7.73 (m, 1H). Elemental analysis calcd (%) for C_10_H_6_N_4_O_2_: C 56.08, H 2.82, N 26.16; found: C 56.04, H 2.85, N 26.12.

### 4.4. Naphtho[2,3-g]pteridine-2,4(1H,3H)-dione *(**2**)*

Compound **2** was synthesized according to the General Procedure, Approach A. Reaction of diamine **B** (0.402 g, 2.53 mmol) with alloxan monohydrate (0.402 g, 2.53 mmol) and boric acid (0.159 g, 2.53 mmol) provided compound **2** as a red solid (yield 0.517 g, 73%). M.p. > 330 °C (lit. 385 °C at 0.001 torr, decomp.) [70]. ^1^H-NMR (DMSO-*d*_6_, TMS): δ 11.93 (s, 1H), 11.73 (s, 1H), 8.90 (s, 1H), 8.52 (s, 1H), 8.26 (d, *J* = 4.8 Hz, 1H), 8.19 (d, *J* = 4.8 Hz, 1H), 7.70–7.65 (m, 1H), 7.63–7.58 (m, 1H). Elemental analysis calcd (%) for C_14_H_8_N_4_O_2_: C 63.64, H 3.05, N 21.20; found: C 63.61, H 2.99, N 21.25.

### 4.5. 8,9-Difluoronaphtho[2,3-g]pteridine-2,4(1H,3H)-dione *(**3**)*

Compound **3** was synthesized according to the General Procedure, Approach A. Reaction of diamine **C** (0.499 g, 3.47 mmol) with alloxan monohydrate (0.561 g, 3.47 mmol) and boric acid (0.211 g, 3.47 mmol) provided compound **3** as a light purple solid (yield 0.504 g, 58%). M.p. 310–311 °C (lit. 310–312 °C) [46]. ^1^H-NMR (DMSO-*d*_6_, TMS): δ 12.01 (s, 1H), 11.78 (s, 1H), 8.29 (dd, *J* = 10.9, 8.6 Hz, 1H), 8.00 (dd, *J* = 11.4, 8.2 Hz, 1H). Elemental analysis calcd (%) for C_10_H_4_F_2_N_4_O_2_: C 48.01, H 1.61, N 22.04; found: C 48.07, H 1.68, N 22.09.

### 4.6. 2,4-Dioxo-1,2,3,4-tetrahydropteridine-6,7-dicarbonitrile *(**4**)*

Compound **4** was synthesized according to the General Procedure, Approach A. Reaction of diamine **D** (0.500 g, 4.63 mmol) with alloxan monohydrate (0.740 g, 4.63 mmol) and boric acid (0.291 g, 4.63 mmol) provided compound **4** as a light grey solid (yield 0.319 g, 32%). M.p. > 330 °C. ^1^H-NMR (DMSO-*d*_6_, TMS): δ 12.87 (br s, 1H), 12.17 (s, 1H). ^13^C-NMR (DMSO-*d*_6_, TMS): δ 158.55, 149.98, 149.35, 134.72, 131.41, 125.39, 114.09, 113.55. Elemental analysis calcd (%) for C_8_H_2_N_6_O_2_: C 44.87, H 0.94, N 39.25; found: C 48.84, H 1.02, N 39.21.

### 4.7. Pyreno[4,5-g]pteridine-11,13(10H,12H)-dione *(**5**)*

Compound **5** was synthesized according to the General Procedure, Approach A. Reaction of diamine **E** (0.270 g, 1.16 mmol) with alloxan monohydrate (0.186 g, 1.16 mmol) and boric acid (0.072 g, 1.16 mmol) provided compound **5** as a yellow solid (yield 0.293 g, 75%). M.p. > 330 °C. ^1^H-NMR (DMSO-*d*_6_, TMS): δ 12.19 (s, 1H), 11.82 (s, 1H), 9.22–9.14 (m, 2H), 8.48 (d, *J* = 7.5 Hz, 1H), 8.39 (d, *J* = 7.5 Hz, 1H), 8.26–8.12 (m, 4H). Elemental analysis calcd (%) for C_20_H_10_N_4_O_2_: C 71.00, H 2.98, N 16.50; found: C 71.08, H 2.95, N 16.44. EI/MS (*m*/*z*): 338.080. Found 338.081 (100). IR (neat, cm^−1^): 3182, 3064, 2937, 2825, 1720, 1689, 1570, 1555, 1447, 1422, 1385, 1364, 1304, 1279, 1234, 1176, 1163, 1033, 928, 859, 829, 812, 752, 718, 653, 617, 590, 525, 487, 459, 445, 434.

### 4.8. Pteridino[6,7-b]phenazine-2,4(1H,3H)-dione *(**6***)

Compound **6** was synthesized according to the General Procedure, Approach A. Reaction of diamine **F** (0.101 g, 0.480 mmol) with alloxan monohydrate (0.077 g, 0.480 mmol) and boric acid (0.030 g, 0.480 mmol) provided compound **6** as a dark purple solid (yield 0.080 g, 57%). M.p. > 330 °C. Elemental analysis calcd (%) for C_16_H_8_N_6_O_2_: C 60.76, H 2.55, N 26.57; found: C 60.74, H 2.52, N 26.50.

### 4.9. General Procedure for the Preparation of the Non-Fused Flavins ***7**–**11*** by Approach B

5,6-Diaminouracil sulphate (1.31 mmol, 1 eq.) was suspended in water (35 mL). The mixture was made alkaline using a methanolic solution of NaOH (0.65M, 3 eq.) and heated to 90 °C for 15 min. A solution of diketone (1.96 mmol, 1.5 eq.) in a mixture of THF (40 mL) and water (15 mL) was added and the mixture was stirred at 90 °C for 45 min. The reaction mixture was acidified glacial acetic acid (15 mL) and heated at 90 °C for another 20 h. The reaction mixture was subsequently concentrated to approximately 1/3 of the volume, cooled in the freezer and the as-obtained suspension was filtered and the solid fraction was washed with cold water (30 mL) and cold ethanol (20 mL) to yield the crude flavin derivative. The crude product was purified by digestion in boiling methanol. The digested flavin derivative was further subjected to vacuum sublimation to yield the final target compound.

### 4.10. Phenanthro[9,10-g]pteridine-11,13(10H,12H)-dione *(**7**)*

Compound **7** was synthesized according to the General Procedure, Approach B. Reaction of diketone **G** (0.817 g, 3.92 mmol) with 5,6-diaminouracil sulphate (1.00 g, 2.62 mmol) provided compound **7** as a yellow solid (yield 0.304 g, 37%). M.p. > 330 °C. ^1^H-NMR (DMSO-*d*_6_, TMS): δ 12.21 (s, 1H), 11.84 (s, 1H), 9.09–8.99 (m, 2H), 8.91–8.81 (m, 2H), 7.98–7.90 (m, 1H), 7.89–7.80 (m, 3H). Elemental analysis calcd (%) for C_8_H_2_N_6_O_2_: C 68.79, H 3.21, N 17.83; found: C 68.66, H 3.11, N 17.75.

### 4.11. 6,7-Diphenylpteridine-2,4(1H,3H)-dione *(**8**)*

Compound **8** was synthesized according to the General Procedure, Approach B. Reaction of diketone **H** (0.577 g, 2.75 mmol) with 5,6-diaminouracil sulphate (0.700 g, 1.83 mmol) provided compound **8** as a bright grey solid (yield 0.262 g, 30%). M.p. 321–323 °C (lit. 320–325 °C) [43]. ^1^H-NMR (DMSO-*d*_6_, TMS): δ 12.06 (s, 1H), 11.73 (s, 1H), 7.44–7.29 (m, 10H). Elemental analysis calcd (%) for C_18_H_12_N_4_O_2_: C 68.35, H 3.82, N 17.71; found: C 68.24, H 3.74, N 17.62.

### 4.12. 6,7-Di(pyridin-2-yl)pteridine-2,4(1H,3H)-dione *(**9**)*

Compound **9** was synthesized according to the General Procedure, Approach B. Reaction of diketone **I** (0.416 g, 1.96 mmol) with 5,6-diaminouracil sulphate (0.500 g, 1.31 mmol) provided compound **9** as a bright beige solid (yield 0.105 g, 25%). M.p. 277–288 °C. ^1^H-NMR (DMSO-*d*_6_, TMS): δ 12.11 (s, 1H), 11.73 (s, 1H), 8.31 (d, *J* = 4.5 Hz, 1H), 8.24 (d, *J* = 4.5 Hz, 1H), 7.92 (tdd, *J* = 7.6, 4.2, 1.7 Hz, 2H), 7.79 (dd, *J* = 14.5, 7.8 Hz, 2H), 7.38 (ddd, *J* = 7.5, 4.8, 1.1 Hz, 1H), 7.31 (ddd, *J* = 7.4, 4.8, 1.2 Hz, 1H). Elemental analysis calcd (%) for C_16_H_10_N_6_O_2_: C 60.38, H 3.17, N 24.40; found: C 60.29, H 3.08, N 24.48.

### 4.13. 6,7-Di(furan-2-yl)pteridine-2,4(1H,3H)-dione *(**10**)*

Compound **10** was synthesized according to the General Procedure, Approach B. Reaction of diketone **J** (0.373 g, 1.96 mmol) with 5,6-diaminouracil sulphate (0.500 g, 1.31 mmol) provided compound **10** as a yellow solid (yield 0.168 g, 43%). Purification of the crude product also involved flash column chromatography with gradient elution (EtOAc → EtOAc/MeOH 50/50 → MeOH) followed by vacuum sublimation. M.p. 285–286 °C (lit. 285–287 °C) [47]. ^1^H-NMR (DMSO-*d*_6_, TMS): δ 12.05 (s, 1H), 11.65 (s, 1H), 7.95 (d, *J* = 1.0 Hz, 1H), 7.85 (d, *J* = 1.0 Hz, 1H), 6.78-6.75 (m, 1H), 6.72–6.68 (m, 2H), 6.55 (d, *J* = 3.1 Hz, 1H). Elemental analysis calcd (%) for C_14_H_8_N_4_O_4_: C 56.76, H 2.72, N 18.91; found: C 56.69, H 2.99, N 19.01.

### 4.14. 6,7-Di(thiophen-2-yl)pteridine-2,4(1H,3H)-dione *(**11**)*

Compound **11** was synthesized according to the General Procedure, Approach B. Reaction of diketone **K** (0.654 g, 2.94 mmol) with 5,6-diaminouracil sulphate (0.750 g, 1.96 mmol) provided compound **11** as a yellow solid (yield 0.264 g, 40%). M.p. > 330 °C (lit. 355–357 °C) [47]. ^1^H-NMR (DMSO-*d*_6_, TMS): δ 12.02 (s, 1H), 11.69 (s, 1H), 7.84 (d, *J* = 4.9 Hz, 1H), 7.79 (d, *J* = 4.9 Hz, 1H), 7.22 (d, *J* = 3.5 Hz, 1H), 7.18–7.07 (m, 3H). Elemental analysis calcd (%) for C_14_H_8_N_4_O_2_S_2_: C 51.21, H 2.46, N 17.06, S 19.53; found: C 51.27, H 2.51, N 17.01, S 19.61.

### 4.15. Optical Measurements

Absorption spectra of solution were measured using a Varian Cary Probe 50 UV-Vis spectrophotometer (Agilent Technology, Santa Clara, CA, USA). The measured spectra were processed using the CaryWinUV software. The absorption spectra were corrected to a baseline, which was determined by measurement of absorption spectra of pure solvent. Solutions were studied in Hellma QS quartz cuvette with an optical path of 10 mm. Solution concentrations of sublimed materials for optical measurements were adjusted to a level of light absorption less than 1. Concentration of all compounds/samples was 1 × 10^−5^ mol·L^−1^. All optical characteristics were measured in ambient air. Excitation and emission spectra of solution were measured using a Horiba Fluorolog (Horiba Jobin Yvon, Kyoto, Japan). The measured spectra were processed using the FluorEssence software. Samples were excited at absorption maxima and varied according to the material.

### 4.16. Electrochemical Measurements

In order to determine the energetic levels for each compound we have conducted electrochemical experiments. Cyclic voltammetry for the materials under investigation was performed using a Jaissle Potentiostat-Galvanostat IMP 88 PC (Jaissle Elektronik GmbH, Waiblingen, Germany). The electrochemical setup consisted of 3-electrode system. A Cr-Au coated (5/80 nm respectively) glass slide coated with the respective small molecule served as the working electrode while a Pt plate was used as the counter electrode. A silver wire coated with silver chloride was used as the quasi reference electrode. Electrolyte solution contained 0.1 M TBAPF_6_ in acetonitrile and the potential was cycles between −1.2 V and 1.2 V vs. Ag/AgCl quasi-reference electrode. The potentials were calibrated internally against ferrocene/ferrocenium couple (Fc/Fc^+^) and the following formula is used to estimate the HOMO and LUMO levels:

E_HOMO_ = −(4.75 eV + E_ox_^onset^ vs. NHE) (1)

E_LUMO_ = −(4.75 eV + E_red_^onset^ vs. NHE) (2)

### 4.17. DFT Calculations

Molecular structures were simulated using the Gaussian 09 software package [71], for all the compounds presented in the paper the electronic structures of the above-mentioned systems were optimized using the 6-311G** split-valence polarized triple-ζ basis set was combined with Becke’s three-parameter hybrid functional with the Lee, Yang, and Parr correlation functional (B3LYP). Absorption spectra were calculated using TD-DFT procedure for 10 lowest excited states.

## Figures and Tables

**Table 1:** Main optical parameters of the compounds.

#	ABS_max_, nm	ABS_max_*, nm	E_g_(opt), eV	E_g_*(opt), eV	ΔE_Stoke’s_, eV	ΔE_Stoke’s_, eV
**1**	386	413	3.0	2.6	0.3	0.6
**2**	390	450	2.9	2.4	(0.5)	0.7
**3**	390	450	3.1	2.5	0.6	0.4
**4**	334	425	3.1	2.6	0.8	0.5
**5**	434	455	2.7	2.5	(0.1)	0.3
**6**	356	417	3.3	2.7	0.4	0.3
**7**	410	448	3.0	2.6	0.1	0.3
**8**	363	410	3.2	2.8	(0.6)	0.6
**9**	363	407	3.2	2.7	0.4	0.6
**10**	358	419	3.1	2.6	(0.6)	0.6
**11**	393	477	2.7	2.4	(0.6)	0.3

ABS_max_, absorption maximum of the short-wavelength chromophore; ABS_max_*, absorption maximum of the long-wavelength chromophore (if applicable); E_g_(opt) optical band gap of the short-wavelength chromophore; E_g_*(opt) optical band gap of the long-wavelength chromophore (if applicable); ΔE_Stoke’s_—Stoke’s shift. Values in brackets are approximate.

**Table 2:** Dihedral rotation angle between the alloxazine/isoalloxazine core and the aromatic rings.

	Alloxazine		Isoalloxazine	
#	α_1_,°^(pos7)^	α_2_,°^(pos6)^	α_3_,°^(pos7)^	α_4_,°^(pos6)^
**8**	34.4	36.6	49.5	32.7
**9**	31.5	31.7	54.5	18.5
**10**	14.3	19.6	31.0	15.3
**11**	17.7	27.2	88.6	2.1

α_1_,° dihedral rotation angle in the X position, α_2_,° dihedral rotational angle in the Y position.

**Table 3:** HOMO and LUMO levels obtained by the electrochemical measurements.

Compound	HOMO (eV)	LUMO (eV)
**1**	−6.30	−4.20
**2**	n.a.	−4.80
**3**	−6.10	−4.30
**4**	−6.30	−4.00
**5**	−6.50	−3.90
**6**	−6.20	−4.50
**7**	n.a.	−4.05
**8**	n.a.	−3.90
**9**	n.a.	−3.93
**10**	n.a.	−4.00
**11**	−6.50	−4.10
